# Moderately differentiated colorectal adenocarcinoma as a lymph node metastatic phenotype: comparison with well differentiated counterparts

**DOI:** 10.1186/1471-2482-10-8

**Published:** 2010-03-08

**Authors:** Shozo Yokoyama, Katsunari Takifuji, Tsukasa Hotta, Kenji Matsuda, Toshiji Tominaga, Yoshimasa Oku, Toru Nasu, Koichi Tamura, Junji Ieda, Hiroki Yamaue

**Affiliations:** 1 Second Department of Surgery, Wakayama Medical University, School of Medicine, 811-1 Kimiidera, Wakayama 641-8510, Japan

## Abstract

**Background:**

The differences between the metastatic property of moderately (Mod) and well (Wel) differentiated colorectal adenocarcinoma remain unclear. Since Mod is unable to form complete acini, therefore an epithelial-mesenchymal transition (EMT) can occur in that structure. Herein, we hypothesized that Mod metastasizes more easily than the Wel counterparts.

**Methods:**

The medical records of 283 consecutive patients with Mod (n = 71) or Wel (n = 212) who underwent surgery were reviewed between January 1, 2001, and December 31, 2003, for actual 5-year overall survival. We examined the differences between the clinicopathological characteristics of the Mod and the Wel groups.

**Results:**

The lymph node involvement (*p *< 0.0001), lymphatic permeation, venous permeation, depth of invasion, liver metastasis, and carcinomatous peritonitis were significantly higher in the Mod group in comparison to the Wel group. The independent risk factors by a logistic regression analysis for lymph node involvement were as follows: lymphatic permeation, liver metastasis, and Mod (*p *= 0.0291, Relative Risk of 1.991: 95% Confidence Interval: 1.073-3.697). A Kaplan-Meier survival curve showed that Mod had a trend towards a poor survival (*p *= 0.0517).

**Conclusion:**

Mod metastasizes to the lymph nodes more easily in comparison to Wel. Therefore, patients with Mod may be considered the existence of lymph node involvement.

## Background

The classification of colorectal cancer such as the TNM classification and Duke's classification do not include the type of histology. Both poorly differentiated adenocarcinoma (Por) [1,2] and mucinous carcinoma (Muc) [3] have a worse prognosis with higher rates of metastasis. However, the histological type of most colorectal cancers are Wel or Mod, and Por and Muc occur in only a small population [4-6]. Since most clinicians believe that Mod is recognized equally well as Wel, therefore there have been no reports comparing the characteristics of Mod in comparison to Wel. In this study, we evaluated whether Mod has a higher rate of metastasis in comparison to Wel, and verified that the histological difference is indicative of the metastatic status and the phenotype of colorectal cancer.

## Methods

Two hundred and eighty three patients, 170 males and 113 females of 26 to 91 years of age were included in this study between January 1, 2001, and December 31, 2003, for actual 5-year overall survival. All of them underwent appropriate colorectal surgery with lymph node resection, and received appropriate chemotherapy for metastatic colorectal cancer. All of the surgical specimens were subjected to a histological analysis by experienced pathologists. The patients were divided into four distinct histological groups as follows: Wel, Mod, Muc, and Por. In classifying the carcinoma, based on the least differentiated component, not including the leading front of invasion, the predominant pattern of the tumor was selected to represent its histological type and grade. In order to compare the differences between Mod and Wel in this study, the patients with Muc and Por were excluded. The Mod group included 71 patients, and the Wel group included 212 patients. The lymph node involvement (positive or negative), the depth of invasion (T1T2 or T3T4), liver metastasis (positive or negative), lung metastasis (positive or negative), carcinomatous peritonitis (positive or negative), lymphatic permeation (positive or negative), venous permeation (positive or negative), gender (female or male), and age (<60 or 60<) were evaluated to assess any differences between the Mod and Wel groups. A univariate and multivariate analysis of data were carried out by using the StatView 5.0 statistical analysis software. The Chi-square test was used to detect differences between the groups. A logistic regression analysis was conducted for the multivariate analysis. All tests were considered significant at *p *< 0.05. We evaluated the overall survival by the Kaplan-Meier method. The study received the approval from Wakayama Medical University Ethics Committee, and was in compliance with the Helsinki Declaration.

## Results

**Figure 1:**
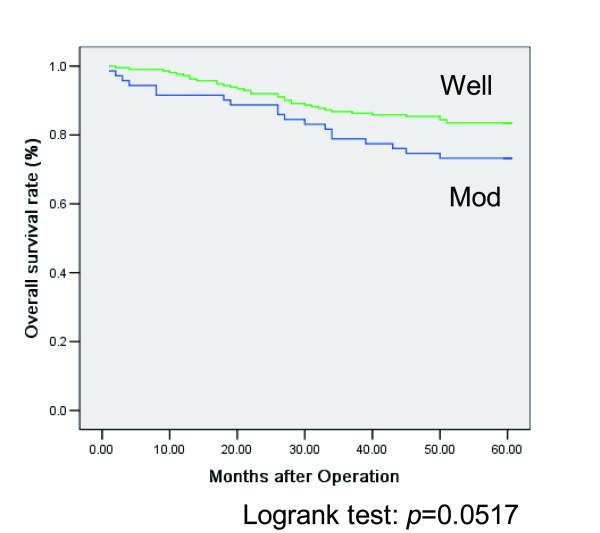
**The overall survival rate of the 71 patients with Mod and the 212 patients with Wel**. Logrank test: *p *= 0.0517. Mod: Moderately differentiated adenocarcinoma; Wel: Well differentiated adenocarcinoma.

A univariate analysis of the histological type showed that the lymph node involvement (*p *< 0.0001), lymphatic permeation (*p *= 0.0001), venous permeation (*p *= 0.0003), depth of invasion (*p *= 0.0018), liver metastasis (*p *= 0.0096), and peritonitis carcinomatosa (*p *= 0.0143) were significantly higher in the Mod group (Table 1) in comparison to the Wel group. Thereafter, we performed a logistic regression analysis of the factors that were found to be significant by the univariate analysis. A multivariate analysis of the lymph node involvement indicated that the independent predictive factors were lymphatic permeation (*p *= 0.0127, Relative Risk 2.650: 95% Confidence Interval 1.232-5.704), liver metastasis (*p *= 0.0240, 2.804: 1.145-6.867), and the histological type of tumor (Mod) (*p *= 0.0291, 1.991: 1.073-3.697) (Table 2). A multivariate analysis of lymphatic permeation showed that the independent predictive factors were venous permeation (*p *< 0.0001, 4.713: 2.393-9.281), depth of invasion (*p *= 0.0006, 3.571: 1.325-6.161), lymph node involvement (*p *= 0.0074, 2.857: 1.073-3.697), and the histological type of tumor (*p *= 0.0390, 2.828: 1.054-7.590) (Table 3). A multivariate analysis of venous permeation, depth of invasion, liver metastasis, and peritonitis carcinomatosa showed that there were no associations between any of these factors and the histological type. A univariate and multivariate analysis showed that Mod was an independent factor for lymph node involvement and lymphatic permeation. The Kaplan-Meier survival curve showed that patients with the Mod histology had a shorter survival (*p *= 0.0517) (Figure 1).

**Table 1:** Comparison between Mod*^1 ^and Wel*^2 ^by a univariate analysis

	Mod (n = 71)	Well (n = 212)	P value
Lymph node involvement	44(62.0%)	74(34.9%)	<0.0001
Lymphatic permeation	65(91.5)	146(68.9)	0.0001
Venous permeation	59(83.1)	126(59.4)	0.0003
Depth of invasion (T3T4)	66(93.0)	161(75.9)	0.0018
Liver metastasis	15(21.1)	20(9.4)	0.0096
Carcinomatous peritonitis	7(9.9)	6(2.8)	0.0143
Age (60<)	52(73.2)	142(67.0)	0.3256
Gender (female)	29(40.8)	84(39.6)	0.8556
Lung metastasis	1(1.4)	3(1.4)	0.9967

*1 Mod: Moderately differentiated adenocarcinoma;

*2 Wel: Well differentiated adenocarcinoma

**Table 2:** A multivariate analysis of the lymph node involvement

	P value	RR*^1^(95% CI*^2^)
Lymphatic permeation	0.0127	2.650 (1.232-5.704)
Liver metastasis	0.0240	2.804 (1.145-6.867)
Mod differentiated * ^3^	0.0291	1.991 (1.073-3.697)

*1 RR, Relative Risk; *2 95%CI, 95% Confidence Interval;

*3 Mod differentiated: Moderately differentiated adenocarcinoma

**Table 3:** A multivariate analysis of the lymphatic Permeation

	P value	RR*^1^(95% CI*^2^)
Venous permeation	<0.0001	4.713 (2.393-9.281)
Depth of invasion	0.0006	3.571 (1.325-6.161)
Lymph node involvement	0.0074	2.857 (1.073-3.697)
Mod differentiated * ^3^	0.0390	2.828 (1.054-7.590)

*1 RR, Relative Risk; *2 95%CI, 95% Confidence Interval

*3 Mod differentiated: Moderately differentiated adenocarcinoma

## Discussion

Most surgeons believe that the characteristics of Mod are similar to Wel, therefore patients with the Mod histology have been treated similarly to patients with the Wel histology. Our data indicated that Mod is more likely to metastasize to the lymph node in comparison to Wel. A multivariate analysis of lymph node metastasis showed that the histological type is ranked third after lymphatic permeation and liver metastasis. The depth of invasion was not an independent marker for lymph node metastasis. This suggested that the histological type including Mod is more important than the depth of invasion. Whereas, a multivariable analysis for lymphantic permeation revealed that histological type is fourth rank after venous permeation, depth of invasion, and lymph node involvement. It indicated that histological type is also important for metastasis to lymphatic vessels. Our results mean that surgeons may consider appropriate surgery with lymph node resection for Mod, if they could have histological information by biopsy or total biopsy such as endoscopic submucosal dissection.

The patients with Wel or Mod underwent surgery with lymph node resection and received appropriate chemotherapy for lymph node (Stage III) and hematogenous metastatic (Stage IV) colorectal cancer. Our results showed that Mod is correlated with lymph node metastasis, therefore the more number of patients with Mod received chemotherapy than Wel. However, our data indicated that the patients with Mod have a trend towards a poorer prognosis in comparison to the patients with Wel. It could be that Stage II colorectal cancer with Mod has micrometastasis, or that Mod is resistant to current chemotherapy. The treatment of patients with Mod, especially for Stage II, by adjuvant chemotherapy may be recommended.

What is the difference between Mod and Wel? Well differentiated adenocarcinoma has a well-constructed structure, namely well formed acini and tubes. However, moderately differentiated adenocarcinoma are characterized by a collapsed structure such as the cribriform pattern [7]. It is postulated that these morphological changes are indicative of an epithelial-mesenchymal transition, and therefore it might be thought that Mod is derived from Wel by the induction of EMT [8]. In addition, EMT was reported to play an essential role in tumorigenesis [9], invasion [8] and metastasis [10]. If Mod is derived from Wel, then Mod may be a metastatic phenotype of Well. Further cellular and molecular examinations are required to confirm this hypothesis.

To some extent, the differences between Mod and Wel have been reported by others [2,6,11]. However, we are the first one to report that Mod is more likely to metastasize than Wel, and that the patients with Mod have a trend towards a poorer prognosis than the patients with Wel. This confirms that the characteristics of Mod have been erroneously thought of as similar to Wel. In this study, we have clearly shown that Mod has a greater lymph node metastatic phenotype than Wel. Further studies to characterize Mod are required in order to understand the mechanism of metastasis and the morphological changes of the cancer tissue.

## Conclusion

The differences in the lymph node metastatic property of Mod in comparison to Wel has been clearly demonstrated in this study. The patients with stage II moderately differentiated colorectal cancer may therefore be treated carefully with a lymph node resection and adjuvant chemotherapy. The mechanism of the morphological changes between Mod and Wel is required for further investigation.

## Competing interests

The authors declare that they have no competing interests.

## Authors' contributions

SY participated in the design of the study, performed statistical analysis and drafted the manuscript. KT participated in its design and coordination. TH helped to draft the manuscript. KM helped to draft the manuscript. TT helped in the revision of the article. YO performed the surgery. TN performed the surgery. KT performed the surgery. JI performed the surgery. HY helped in the revision of the article, and gave approval for the final write up. All authors read and approved the final manuscript.

## Pre-publication history

The pre-publication history for this paper can be accessed here:

http://www.biomedcentral.com/1471-2482/10/8/prepub
